# In Vitro Zika Virus Infection of Human Neural Progenitor Cells: Meta-Analysis of RNA-Seq Assays

**DOI:** 10.3390/microorganisms8020270

**Published:** 2020-02-17

**Authors:** Rossella Gratton, Paola Maura Tricarico, Almerinda Agrelli, Heverton Valentim Colaço da Silva, Lucas Coêlho Bernardo-Menezes, Sergio Crovella, Antonio Victor Campos Coelho, Ronald Rodrigues de Moura, Lucas André Cavalcanti Brandão

**Affiliations:** 1 Department of Advanced Translational Microbiology, Institute for Maternal and Child Health IRCCS Burlo Garofolo, Via dell’Istria 65/1, 34137 Trieste, Italy; tricaricopa@gmail.com (P.M.T.); crovelser@gmail.com (S.C.); 2 Department of Medical, Surgical and Health Sciences, University of Trieste, Strada di Fiume 447, 34129 Trieste, Italy; 3 Laboratory of Immunopathology Keizo Asami (LIKA), Federal University of Pernambuco (UFPE), Av. Prof. Moraes Rego, 1235 Cidade Universitária, 50670-901 Recife, Brazil; almerindaapimentel@hotmail.com (A.A.); neto_pe6@live.com (H.V.C.d.S.); lucascoelhobernardo.lb@gmail.com (L.C.B.-M.); lucabrand@gmail.com (L.A.C.B.); 4 Department of Pathology-Federal University of Pernambuco (UFPE), Av. Prof. Moraes Rego, 1235-Cidade Universitária, 50670-901 Recife, Brazil; ronaldmoura1989@gmail.com; 5 Department of Genetics-Federal University of Pernambuco (UFPE), Av. Prof. Moraes Rego, 1235-Cidade Universitária, 50670-901 Recife, Brazil; 6 Department of Molecular Biology-Federal University of Paraíba (UFPB), Campus I-Lot.-Cidade Universitária, 58051-900 João Pessoa, Brazil; antoniocampos@dbm.ufpb.br

**Keywords:** congenital Zika syndrome, transcriptomics, genomics, apoptosis, gene ontology, pathway analysis, stem cells

## Abstract

The Zika virus (ZIKV) is an emergent arthropod-borne virus (arbovirus) responsible for congenital Zika syndrome (CZS) and a range of other congenital malformations. Evidence shows that ZIKV infects human neural progenitor cells (hNPCs) in the fetal brain, prompting inflammation and tissue damage/loss. Despite recent advances, little is known about the pathways involved in CZS pathogenesis. We performed a meta-analysis, gene ontology (GO), and pathway analysis of whole transcriptome studies with the aim of clarifying the genes and pathways potentially altered during hNPCs infection with ZIKV. We selected three studies (17 samples of infected hPNCs compared to hPNCs uninfected controls) through a systematic search of the Gene Expression Omnibus (GEO) database. The raw reads were trimmed, counted, and normalized. Next, we performed a rank product meta-analysis to detect consistently differentially expressed genes (DEGs) in these independent experiments. We detected 13 statistically significant DEGs. GO ontology and reactome analysis showed an enrichment of interferon, pro-inflammatory, and chemokines signaling and apoptosis pathways in ZIKV-infected cells. Moreover, we detected three possible new candidate genes involved in hNPCs infection: *APOL6*, *XAF1*, and *TNFRSF1*. Our results confirm that interferon (IFN) signaling dominates the ZIKV response, and that a crucial contribution is given by apoptotic pathways, which might elicit the CZS phenotype.

## 1. Introduction

The Zika virus (ZIKV) is an arthropod-borne virus (arbovirus), member of the *Flaviviridae* family and of the *Flavivirus* genus, transmitted by *Aedes* genus mosquitoes [1]. In 2015, during the outbreak in Brazil, ZIKV was correlated for the first time to neonatal microcephaly [2] and to a variety of other congenital malformations, especially of neurological origin, collectively known as congenital Zika syndrome (CZS) [3].

CZS is characterized by a spectrum of congenital malformations associated with ZIKV infection during embryonic development [4]. The most commonly reported neurological feature of CZS is microcephaly, a condition characterized by a head circumference ≥2 standard deviations below the mean for sex and gestational age at birth, although other neurological abnormalities, including brainstem dysfunction, absence of swallowing reflex, and polymalformative syndromes, may also be present [5]. General features (redundant scalp skin, anasarca, low birth weight, polyhydramnios, and arthrogryposis) and ophthalmological defects (intraocular calcifications, cataract, asymmetrical eye sizes, macular atrophy, optic nerve hypoplasia, iris coloboma, and lens subluxation) have also been reported [6,7,8].

The human central nervous system (CNS) development begins during the third week of embryogenesis [9]. The embryonic brain is basically composed of human neural progenitor cells (hNPCs), progenitor cells that give rise to all of the glial and neuronal cell types that populate the CNS; therefore, the onset of pathogenic processes might cause neuroinflammation and the secretion of immunoregulatory molecules [10]. As a result, these events may trigger cell death mechanisms, leading to an impairment of hNPCs proliferation, growth, and differentiation, and consequently to a defective brain development [11].

Several studies demonstrated that ZIKV infects hNPCs in the fetal brain, prompting inflammation and tissue damage and loss [12,13,14,15]. Despite recent advances in the characterization of the impact of ZIKV infection on embryonic CNS development, it is still necessary to identify which pathways in hNPCs are involved during these pathogenic mechanisms. This gap of knowledge is clearly restrictive for the development of therapeutic approaches that could prevent the severe clinical consequences of the infection.

Transcriptional profiling has provided remarkable opportunities for understanding the relationship between cellular function and metabolic pathways, as well as to define the possible implications of genetic variability and environmental conditions in many tissues and organisms [16]. RNA-sequencing (RNA-Seq) has been widely used over the last decade and has become the main option for these studies [17,18].

In this work, we performed a meta-analysis of whole transcriptome studies, aiming to clarify which genes and cellular networks were up- or downregulated during ZIKV infection in hNPCs. Next, we assessed a comprehensive pathway analysis to predict how the modulation of these genes could affect the outcome of the disease.

## 2. Materials and Methods

### 2.1. Study Search

We used the SRAdb package [19] for R software version 3.6.1 [20] to search for RNA-Seq experiments deposited in the Gene Expression Omnibus (GEO) database related to ZIKV infection in hNPCs that matched the following criteria: only whole transcriptome studies; experiments carried out in patients’ cells (ex vivo) or human cell lines (in vitro); and availability of the raw data (.fastq files) for each sample. The following search terms were used: “ZIKV RNA-Seq” and “ZIKV transcriptome” including studies from 19 July 2015 to 19 July 2019.

### 2.2. RNA-Seq Data Collection, Processing, and Analysis

For all analyzed samples, Raw .fastq files were downloaded and re-processed using the same pipeline analysis. For this purpose, Trimmomatic v0.39 [21] was used to trim adapters and to exclude reads counting less than 25 bases. Then, the remaining reads were mapped on the National Center for Biotechnology (NCBI) human GRCh38 reference genome and sorted by coordinates using STAR aligner [22]. Aligned reads were imported into R software version 3.6.1 [20], together with a .gtf annotation file from the reference genome, using packages *Rsamtools* [23], *GenomicFeatures*, and *GenomicAlignments* [24].

The gene counts were normalized and filtered in order to remove low-expressed genes (i.e., genes expressed in less than three samples and less than two copies). Differentially expressed genes (DEGs) for each study were re-calculated using a Wald test with correction for multiple tests implemented in the *DESeq2* package [25]. Genes with |log2(fold change)| > 1 and false discovery rate (FDR)-adjusted *p*-values < 0.05 were considered to be statistically significant.

### 2.3. Meta-Analysis

The normalized and filtered gene expression dataset, together with the samples’ information relative to the type of treatment (ZIKV-infected or control) and the team that performed the study (Zhang et al., McGrath et al., or Caires-Júnior et al.) were included in the meta-analysis using the *RankProd* package for R software version 3.6.1 [20]. Briefly, the package performs the rank product (RP) and rank sum tests, non-parametric tests that detect consistently differentially expressed genes in independent and replicated experiments. The test ranks expression fold-changes calculated in a pairwise manner among several experimental replicates. Under the null hypothesis (no differentially expressed genes), it “is extremely unlikely to find the same gene at the top of each list [*of ranked fold changes among experiments*] just by chance” [26,27]. We adopted the meta-analysis methodology derived from a previously published study, in which a gene ontology enrichment analysis was also included (see the next section) [28].

### 2.4. Gene Ontology Enrichment Analysis

In parallel with the identification of differentially expressed genes via meta-analysis, we performed a gene ontology enrichment analysis by employing the *GOexpress* package [29], also suitable for R software version 3.6.1 [20]. Briefly, the package scores each gene feature (through a random forest statistical framework) on “its ability to classify samples from different treatments separately, before summarizing this information at the ontology level” [29]. Then, the package queries the ranked genes in the Ensembl gene ontology database and configures the package to assess the statistical significance of the gene ontology ranking via permutation-based calculation of *p*-values (100,000 permutations), representing the probability of seeing at least five genes out of the total number of genes in the list attributed to a particular gene ontology term [30,31]. Then, the genes were ranked from the lowest to highest *p*-value below the limit of *p* < 0.05.

### 2.5. Reactome Pathway Analysis

For each independent study and for the pooled dataset in the meta-analysis, we conducted a pathway analysis, based on the REACTOME database, of the statistically significant DEGs using the *ReactomePA* package [20]. Also, in this case, only the results with false discovery rate (FDR)-adjusted *p*-values < 0.05 were considered significant.

## 3. Results

The search strategy retrieved 30 studies. Three people (R.R.M., H.V.C.S., and L.C.B.-M.) independently reviewed the search hits, from which 10 studies were excluded because only viral genomes were sequenced; four were excluded since they were studies performed in mice; three experiments were eliminated since they involved other animals (mice and *Aedes aegypti* mosquitoes); one study was removed since it was relative to the sequencing of the Chikungunya virus genome; six studies involving other human cell types and three studies based on CNS organoids were also excluded as they contemplated the usage of other cell types beside hNPCs. Finally, the three reviewers agreed that three studies matched the fixed criteria (Table 1) [32,33,34].

The first study, from Zhang et al. (SRAdb id: SRP073493, GSE id: GSE80434) [32], analyzed the differences between ZIKV and Dengue virus (DENV) infection in hNPCs. In the selected research, hNPCs were infected with both African (ZIKVM) and Asian (ZIKVC) lineages to then compare the levels of transcriptional changes, gene function, and protein interactions among the DEGs. We focused our attention only on the comparisons between ZIKVM versus mock treatment and ZIKVC versus mock treatment. Their study highlighted 1345 DEGs between ZIKVM and its mock group and 601 DEGs for the ZIKVC versus mock group comparison.

The work of McGrath et al. [33] (SRAdb id: SRP096367, GSE id: GSE93385) was the second study to be included. It comprised the analysis of hNPC samples derived from three deceased children. Whole transcriptome profiles were compared between infected and non-infected cells of each patient separately. As a result, they detected eight upregulated and four downregulated genes, found to be shared between the samples.

The third study, conducted by Caires-Júnior et al. [34] (SRAdb id: SRP114529, GSE id: GSE102128) described the performance of an RNA-Seq experiment on cells derived from three pairs of discordant phenotypes of CZS dizygotic twins. According to their results, authors identified 64 DEGs, and specifically the *DDIT4L* gene, which plays a crucial role in the mammalian target of rapamycin (mTOR) signaling pathway and emerged as the most relevant one.

Since each study possesses its own approach in terms of handling and statistical procedures of RNA-Seq data, we reprocessed the reads using the same protocols for all samples and performed a meta-analysis. The normalization of expression data resulted in 29,318 features present in all 17 samples. Among those, we identified 13 upregulated genes in virus-infected cells through the rank product method with a percentage of false prediction, pfp < 0.05 and |log2(fold change)| > 1 (Table 2).

The gene ontology analysis identified 847 enriched terms. Due to the stem cell nature of the cells used in the examined experiments, as expected, most of the top-ranked terms included events representative of cell cycle progression (“mitotic spindle midzone assembly”, rank #1, *p* < 0.00001) and cell differentiation (“nervous system development”, #12, *p* < 0.00001, “multicellular organism development”, #15, *p* < 0.00001; “cell differentiation”; #39, *p* = 0.00021). We then selected 20 terms possibly related to ZIKV-infection and response (Table 3), including for example: “response to virus” (#49, *p* = 0.00033), “apoptotic process” (#57, *p* = 0.00042), “viral process” (#64, *p* = 0.0005), “positive regulation of neuron death” (#132, *p* = 0.00239), and “regulation of inflammatory response” (#142, *p* = 0.00266). The complete list of enriched GO terms can be found in the Supplementary Table S1.

From the 13 DEGs that reached our |log2(fold change)| threshold, reactome analysis returned 12 statistically significant pathways in which these genes are involved (Table 4). Briefly, these pathways are related to interferon signaling and response, as well as to interleukin-10, chemokines, and other receptor signaling.

## 4. Discussion

We searched for studies involving hNPCs transcriptome analysis in response to ZIKV infection. We included three available studies and analyzed the gene expression patterns using a meta-analysis with the rank product method. Using a different approach, we detected 13 statistically significant DEGs found to be upregulated in hNPCs infected by ZIKV. No downregulated gene was observed as statistically associated with hNPCs infected by ZIKV. Our goal was to identify the expression pattern during ZIKV infection in hNPCs, primarily in order to highlight potential molecules that could be used as an antiviral barrier, namely restriction factors, and identify which molecules are released during the antiviral response.

Differences amongst the number and the identity of differentially expressed genes found in our meta-analysis, and in the selected studies, individually rely on three main factors. The first aspect to be considered is that each study has its own peculiarities in terms of the analyzed samples and conditions of infection, as highlighted in Table 1. The second aspect depends on the fact that the studies did not apply the same method for the quantitative evaluation of each mRNA. Specifically, the study of Zhang et al. [32] used fragments per kilobase million (FPKM) measurements for mRNA counting and then applied gamma-Poison normalization, while we opted for the usage of a simple count followed by the application of the gamma-Poison normalization. Third, the experimental design among the studies was clearly different. For instance, in the work conducted by McGrath et al. [33], they assessed a pair-wise comparison between infected and non-infected cells from each evaluated brain sample followed by the application of a Venn diagram to verify the differences (or resemblances) between infected and non-infected samples as a whole. Furthermore, the statistical procedures carried out by Caires-Junior et al. [34] were very similar to the ones we employed in our study, although it is important to note that the contexts of application were different since we were assessing them in a meta-analysis context, whereas they made a single study.

Being that the sampled cells were hNPCs, between the identified DEGs, three are known to be involved in tissue development: the NK3 homeobox 1 (*NKX3-1*) and homeobox A2 (*HOXA2*), which encode for homeobox-containing transcription factors and promote animal morphogenesis and tissue differentiation [35,36], and chitinase-3-like protein 1 (*CHI3L1*), which codes for a chitinase-like protein that lost the capability for chitin cleavage, but retained the carbohydrate-binding affinity, and is thought to regulate tissue remodeling and angiogenesis [37]. 

2′-5′-Oligoadenylate synthetase 1 and 3 (*OAS1* and *OAS3*) and 2′-5′-oligoadenylate synthetase like (*OASL*) are related to antiviral responses and were detected as being upregulated in their ZIKV-infected counterparts. They are interferon-inducible genes that bind to double-stranded RNA (dsRNA) and single-stranded RNA (ssRNA) viral genomes and activate the RNase L degradation pathway of viral and cellular RNA, therefore arresting viral production [38]. A recent study demonstrated that while ZIKV ssRNA genome is susceptible to RNase L activity, the virus otherwise efficiently evades the enzymatic activity due to its unique replication factories in the endoplasmic reticulum, which confers a higher resistance to host viral sensors when compared to other *Flaviviruses*, such as DENV, that employ similar assembly strategies [39].

However, the most intriguing evidence is represented by the upregulation of interferon-inducible genes, such as interferon induced with helicase C domain 1 (*IFIH1*) and apolipoprotein L6 (*APOL6*) genes. *IFIH1*, also known as melanoma differentiation-associated protein 5 (*MDA5*), encodes for a pattern recognition receptor that, along with the DDX58 (RIG1) protein, is able to bind dsRNA and ssRNA derived from other *Flaviviruses*, such as DENV, and elicits innate immune responses through type I interferon (IFN-1) production [40]. Moreover, this gene has already been seen to be upregulated in skin cells during ZIKV infection [41].

*APOL6* has been reported to not only promote antiviral responses against some viruses, such as picornaviruses, enteroviruses, and respiratory syncytial virus [42,43], but also to possess pro-apoptotic properties [44]. We could not find any other independent evidence relating the expression of this gene in the context of ZIKV infection.

It is important to note that in our analysis, we identified two other DEGs not yet independently associated with ZIKV infection, to our knowledge: X- linked inhibitor of apoptosis protein associated factor 1 (XAF1) and tumor necrosis factor receptor superfamily member 1A (*TNFRSF1*). *XAF1* is involved in the apoptotic route and it encodes for a protein acting as an antagonist of inhibitors of apoptosis proteins (IAPs), therefore also exerting pro-apoptotic properties. Its expression is also induced by interferon (IFN) signaling and is thought to function as a tumor suppressor [45]. We could not ascertain other studies showing *XAF1* association with ZIKV infection. *TNFRSF1* encodes for a widely expressed protein, which is the main receptor involved in mediating soluble TNFα-induced signaling, therefore exerting a pivotal role in regulating pro-inflammatory and immune responses [46]. *TNFRSF1* has not been reported to be involved with ZIKV infection; nevertheless, other studies highlight its role in the immune defense in the gut mucosa [47].

As previously described in the text, the majority of the identified DEGs mediate antiviral intracellular pathways. However, besides *TNFRSF1*, some other genes involved in inflammatory and immune responses were also upregulated in ZIKV-infected hNPCs: C-C motif chemokine ligand 5 (*CCL5*) and C-X-C motif chemokine ligand 10 and 11 (*CXCL10* and *CXCL11*) [48]. *CCL5*, *CXCL10*, and *CXCL11* have already been shown to be expressed in ZIKV-infected skin cells [41].

Integrating our results in the context of neurogenesis, we suppose that the presence of ZIKV in hNPCs is sensed by interferon induced with helicase C domain 1 (IFIH1), which acts as a restriction viral factor, enabling the cell to activate type 1 interferon apoptosis and inflammatory pathways, as suggested by reactome analysis. Furthermore, the activation of inflammatory processes might increase the production of cytokines and chemokines (*CXCL10* and *CXCL11*) that may play a key role during neurogenesis by regulating the expression of developmental genes. Thus, some neurological CZS symptoms may be due to the development of an interfering inflammatory response during neurogenesis [49].

## 5. Conclusions

**Figure 1:**
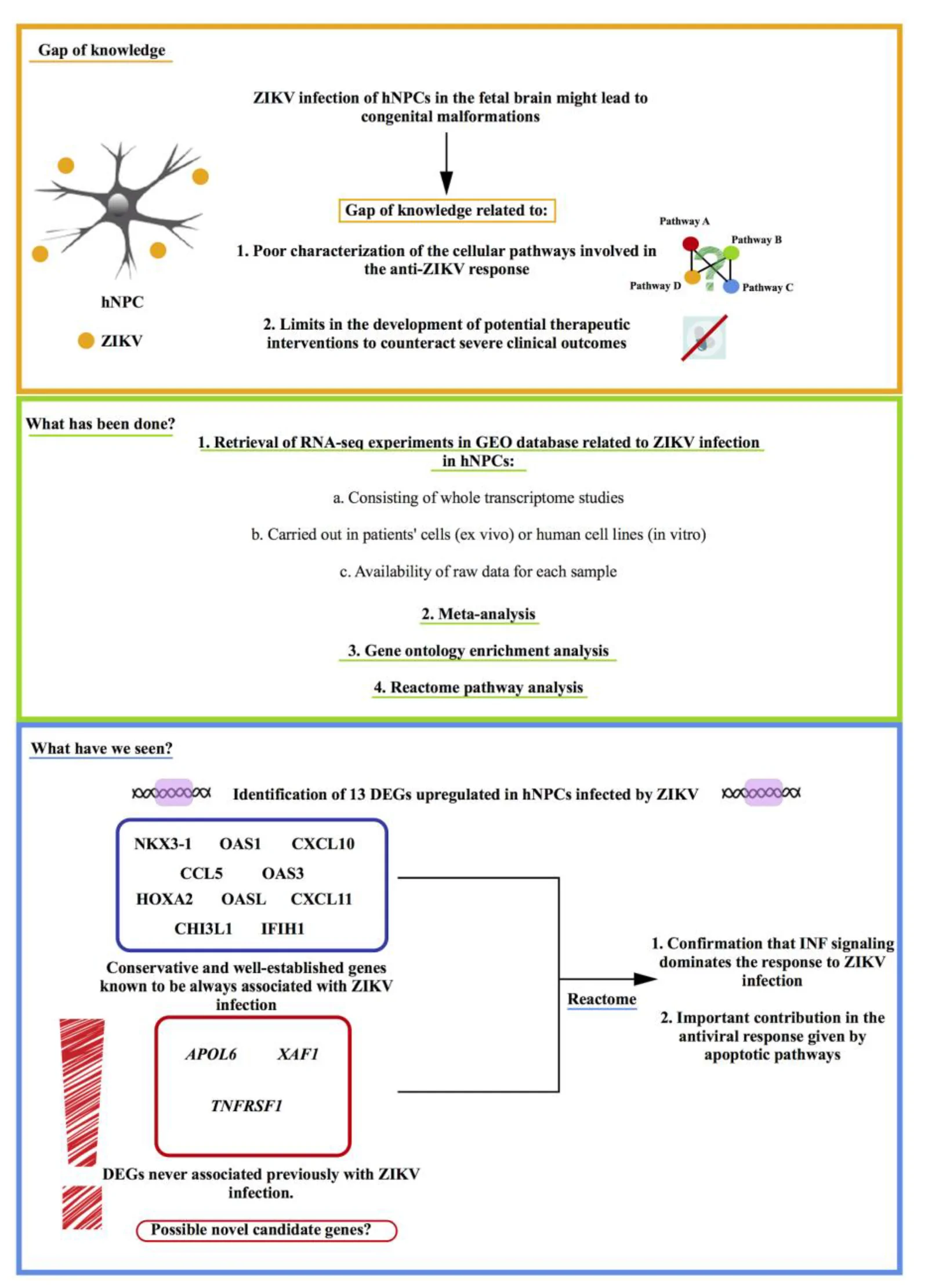
Meta-analysis of RNA-Seq assays for the characterization of congenital Zika syndrome (CZS). ZIKV can infect hNPCs in the fetal brain, possibly leading to congenital malformations. Despite recent advances, the characterization of the main cellular pathways involved in the anti-ZIKV response are yet not fully understood and clearly constitute a limitation for the development of therapeutic approaches that could prevent the severe clinical consequences of the infection. By performing a meta-analysis, gene ontology, and reactome pathway analysis of whole transcriptome studies, we aimed at clarifying the genes and pathways that are potentially altered during hNPCs infection with ZIKV. Our results led to the identification of 13 DEGs found to be upregulated in hNPCs infected by ZIKV. Specifically, we detected three possible new candidate genes, never previously associated to ZIKV-infection, expressed during the antiviral response in infected hPNCs: *APOL6*, *XAF1*, and *TNFRSF1.* Finally, we have confirmed that INF signaling dominates the response against ZIKV infection and that an important contribution is given by apoptotic pathways that might elicit the CZS phenotype.

RNA-Seq has contributed to increase the knowledge relative to the biological and cellular processes involved during infections, especially in the context of an emergent pathogen, such as ZIKV. Meta-analysis of RNA-Seq assays increases the statistical power of samples and we applied this method for re-examining past evidence to better understand the pathogenesis of CZS. The results of our GO enrichment and reactome analysis seem to support the assumption that the cellular antiviral pathways activated in response to ZIKV infection could be responsible for an impaired cellular neurogenesis. As mentioned above, other studies investigating ZIKV infection also support the biological significance of our meta-analysis findings. Moreover, we detected three possible novel candidate genes involved during this antiviral response: *APOL6*, *XAF1*, and *TNFRSF1*. Finally, we confirmed that IFN signaling dominates the cellular response against ZIKV infection, and that under these conditions, an important contribution is given by apoptotic pathways that might elicit the CZS phenotype (Figure 1).

## Figures and Tables

**Table 1:** Detailed information regarding the three selected studies that matched the study criteria. DEGs: differentially expressed genes, hiPSCs (human-induced pluripotent stem cells), MOI (multiplicity of infection), NPCs (neural progenitor cells), RNA-Seq (RNA sequencing), ZIKV (Zika virus).

SRA	Title	Samples	Replicates	Main Results
SRP073493	Molecular Signatures Associated with ZIKV Exposure in Human Cortical Neural Progenitors [32]	Three infected (two with African and one with Asian lineage); two non-infected	Two per sample	The RNA-Seq extraction was gone in 56 hpi for African lineage and 64 hpi for Asian lineage. MOI of 0.2 and 0.4. DEGs include *TP53*.
SRP096367	Differential Responses of Human Fetal Brain Neural Stem Cells to Zika Virus Infection [33]	Three infected with Asian or African lineage; three non-infected	Three per sample	Usage of isolates from Mexico (Asian lineage), Cambodia (Asian lineage), and Senegal strains (African lineage). Following 120 hpi to RNA-Seq extraction. MOI of 0.1 and 1. The DEGs found were *FAS*, *SOX1*, and *TUBB3*.
SRP114529	RNA-seq of hiPSCs-Derived NPCs from Three Pairs of Dizygotic Discordant Twins for Congenital Zika Syndrome [34]	Three infected with Asian lineage; three non-infected	One per sample	Brazilian strain (Asian lineage) used at a MOI of 0.01 and 0.1. RNA-Seq extracted 96 hpi. Indentified DEGs included *DEPDC5*, *GPR108*, *MICAL3*, *OR12D2*, *OR4K5, PHF2*, *SLC6A18*, and *TTC16*.

**Table 2:** Meta-analysis results of three RNA-Seq assays involving human neural stem cells experimentally infected in vitro with Zika virus when compared to control cells, ranked by lowest *p*-values corrected by the percentage of false prediction, pfp (rank product test).

Gene	Rank Product	Fold Change	log2 (Fold Change)	*p*-Value	pfp
		(Control/ZIKV-Infected)			
*OAS1*	334.5	0.228	−2.1329	1.874 × 10^−9^	0.0001
*CXCL10*	350.9	0.3021	−1.7269	2.664 × 10^−9^	0.00004
*OASL*	840.8	0.3739	−1.4193	0.000001	0.0088
*CCL5*	880.8	0.2667	−1.9067	0.000002	0.0095
*CXCL11*	997.5	0.1634	−2.6135	0.000004	0.0153
*TNFRSF1*	1001	0.2218	−2.1727	0.000004	0.0137
*IFIH1*	1085	0.2048	−2.2877	0.000006	0.0205
*OAS3*	1150	0.3442	−1.5387	0.000009	0.0266
*CHI3L1*	1158	0.3843	−1.3797	0.00001	0.0254
*NKX3-1*	1164	0.3466	−1.5287	0.00001	0.0241
*APOL6*	1203	0.1532	−2.7065	0.00001	0.0273
*XAF1*	1251	0.283	−1.8213	0.00002	0.0323
*HOXA2*	1314	0.4441	−1.1710	0.00002	0.0383

**Table 3:** List of 20 gene ontology (GO) terms that were enriched in the meta-analysis of three RNA-Seq assays involving human neural stem cells that were experimentally infected in vitro with Zika virus when compared to control cells (false discovery rate (FDR)-adjusted *p*-values). The complete list of all 847 terms can be found in Supplementary Table S1.

Rank	GO Term	Genes in GO Term	Genes of GO Term Present in Data	Adjusted *p*-Value	GO Term Description
23	GO:0043066	541	526	0.00071	Negative regulation of apoptotic process
49	GO:0009615	109	109	0.00570	Response to virus
57	GO:0006915	693	666	0.00617	Apoptotic process
64	GO:0016032	476	460	0.00662	Viral process
69	GO:0033209	118	117	0.00749	Tumor necrosis factor-mediated signaling pathway
125	GO:1902237	11	11	0.01385	Positive regulation of endoplasmic reticulum stress-induced intrinsic apoptotic signaling pathway
132	GO:1901216	41	41	0.01523	Positive regulation of neuron death
142	GO:0050727	81	81	0.01576	Regulation of inflammatory response
152	GO:0006954	386	372	0.01612	Inflammatory response
257	GO:0006959	56	56	0.02419	Humoral immune response
282	GO:0043065	375	360	0.02500	Positive regulation of apoptotic process
328	GO:0051607	201	195	0.02596	Defense response to virus
546	GO:0042771	29	29	0.03671	Intrinsic apoptotic signaling pathway in response to DNA damage by p53 class mediator
570	GO:0034612	32	32	0.03800	Response to tumor necrosis factor
576	GO:0002523	9	9	0.03854	Leukocyte migration involved in inflammatory response
639	GO:0097194	18	18	0.04256	Execution phase of apoptosis
641	GO:0045089	25	25	0.04259	Positive regulation of innate immune response
683	GO:0002741	8	8	0.04391	Positive regulation of cytokine secretion involved in immune response
704	GO:0002437	15	15	0.04585	Inflammatory response to antigenic stimulus
805	GO:0002827	9	9	0.04832	Positive regulation of T-helper 1 type immune response

**Table 4:** A list of “reactome” pathways that were enriched in the meta-analysis of three RNA-Seq assays involving human neural stem cells experimentally infected in vitro with Zika virus when compared to control cells. FADD/RIP-1 (fas-associated death domain and receptor interacting protein 1), GPCR (G protein-coupled receptor), IFN (interferon), IRF (interferon regulatory factor), NF-kB (nuclear factor kappa B), TRAF3 (tumor necrosis factor receptor-associated factor 3).

Reactome ID	Description	*p*-Value	Adjusted *p*-Value	Gene Symbols
R-HSA-1169410	Antiviral mechanism by IFN-stimulated genes	6.50 × 10^−5^	0.0005	*OAS1/OASL/OAS3*
R-HSA-373076	Class A/1 (Rhodopsin-like receptors)	0.0039	0.0135	*CXCL10/CCL5/CXCL11*
R-HSA-375276	Peptide ligand-binding receptors	0.0008	0.0039	*CXCL10/CCL5/CXCL11*
R-HSA-380108	Chemokine receptors bind chemokines	1.39 × 10^−5^	0.0002	*CXCL10/CCL5/CXCL11*
R-HSA-418594	G alpha (i) signaling events	0.0073	0.0228	*CXCL10/CCL5/CXCL11*
R-HSA-500792	GPCR ligand binding	0.0101	0.0282	*CXCL10/CCL5/CXCL11*
R-HSA-6783783	Interleukin-10 signaling	0.0010	0.0041	*CXCL10/CCL5*
R-HSA-877300	Interferon gamma signaling	9.87 × 10^−5^	0.0005	*OAS1/OASL/OAS3*
R-HSA-909733	Interferon alpha/beta signaling	5.21 × 10^−7^	1.46 × 10^−5^	*OAS1/OASL/OAS3/XAF1*
R-HSA-913531	Interferon Signaling	3.56 × 10^−5^	0.0003	*OAS1/OASL/OAS3/XAF1*
R-HSA-918233	TRAF3-dependent IRF activation pathway	0.0144	0.0336	*IFIH1*
R-HSA-933543	NF-kB activation through FADD/RIP-1 pathway mediated by caspase-8 and -10	0.0127	0.0315	*IFIH1*

## Supplementary Materials

The following are available online at https://www.mdpi.com/2076-2607/8/2/270/s1, Table S1: A complete list of all 847 GO terms that were enriched in the meta-analysis of three RNA-Seq assays involving human neural stem cells.
